# Inhibitor of DNA binding in heart development and cardiovascular diseases

**DOI:** 10.1186/s12964-019-0365-z

**Published:** 2019-05-24

**Authors:** Wenyu Hu, Yanguo Xin, Jian Hu, Yingxian Sun, Yinan Zhao

**Affiliations:** 1 Department of Cardiology, The First Affiliated Hospital of China Medical University, Shenyang, 110001 Liaoning China; 2 Department of Cardiology, West China Hospital of Sichuan University, Chengdu, 610041 Sichuan China; 3 Department of Neurology, The First Affiliated Hospital of China Medical University, Shenyang, 110001 Liaoning China

**Keywords:** Cardiac conduction system, Heart development, Id, Inhibitor of DNA binding

## Abstract

Id proteins, inhibitors of DNA binding, are transcription regulators containing a highly conserved helix-loop-helix domain. During multiple stages of normal cardiogenesis, Id proteins play major roles in early development and participate in the differentiation and proliferation of cardiac progenitor cells and mature cardiomyocytes. The fact that a depletion of Ids can cause a variety of defects in cardiac structure and conduction function is further evidence of their involvement in heart development. Multiple signalling pathways and growth factors are involved in the regulation of Ids in a cell- and tissue- specific manner to affect heart development. Recent studies have demonstrated that Ids are related to multiple aspects of cardiovascular diseases, including congenital structural, coronary heart disease, and arrhythmia. Although a growing body of research has elucidated the important role of Ids, no comprehensive review has previously compiled these scattered findings. Here, we introduce and summarize the roles of Id proteins in heart development, with the hope that this overview of key findings might shed light on the molecular basis of consequential cardiovascular diseases. Furthermore, we described the future prospective researches needed to enable advancement in the maintainance of the proliferative capacity of cardiomyocytes. Additionally, research focusing on increasing embryonic stem cell culture adaptability will help to improve the future therapeutic application of cardiac regeneration.

## Background

The mammalian heart is among the earliest formed organs during development. After 6.5 days of the embryonic period (E6.5), the gastrulation-formed mesoderm moves forward in the embryo and at E7.5 forms the cardiac crescent, which is the precursor to the heart. After the myocardial progenitor cells take up residence within the cardiac mesoderm at E7.5, the cardiac crescent can be divided into two layers according to the differential gene expression: the first and the second heart field. The first heart field consists of cardiomyocytes marked by the cardiac transcription factor Nkx2.5 [1]; it begins to undergo the process of differentiation and is surrounded by undifferentiated precursors from the second heart field. The first heart field gives rise to the left ventricle, part of the atria, and the sinus venosus. The second heart field progenitors gradually migrate into the heart tube, differentiating and giving rise to the right ventricle, the rest of the atria and the outflow tract. With the formation of valves and the intermediate septum inside the heart tube, the primitive atria and ventricle are separated and gradually develop into the mature four-chamber cardiac configuration [2]. Precisely controlled gene expression is essential for normal and effective heart function. During the developmental process of the heart, basic helix-loop-helix (bHLH) transcription factors, such as Hey1/2 [3, 4], Hand1/2 [5, 6], Mesp1/2 [7, 8], and Twist1 [9], direct the expression of cardiac genes, thereby playing crucial roles in the regulation of cardiac chamber septation as well as outflow tract and valve morphogenesis. bHLH factors induce transcription as homodimeric or heterodimeric complexes by binding to the target gene at a specific recognition motif in the promoter region, named E-box (CANNTG) or N-box (CACNAG) [10]. Inhibitor of DNA binding (Id) proteins belong to the HLH family of transcription factors, which have an HLH domain but lack a DNA-binding one, thus functioning as negative regulators of bHLH factors through the formation of non-functional HLH-bHLH heterodimers. As transcriptional regulators, four members of the Id family are involved in many pivotal aspects of heart development by competitively forming non-functional heterodimers with other ubiquitously expressed bHLH factors. In this review, we will outline the regulatory role of the Id family components in heart development and cardiovascular diseases and discuss some unsolved questions about their developmental functions.

### Overview of the id family

**Fig. 1:**
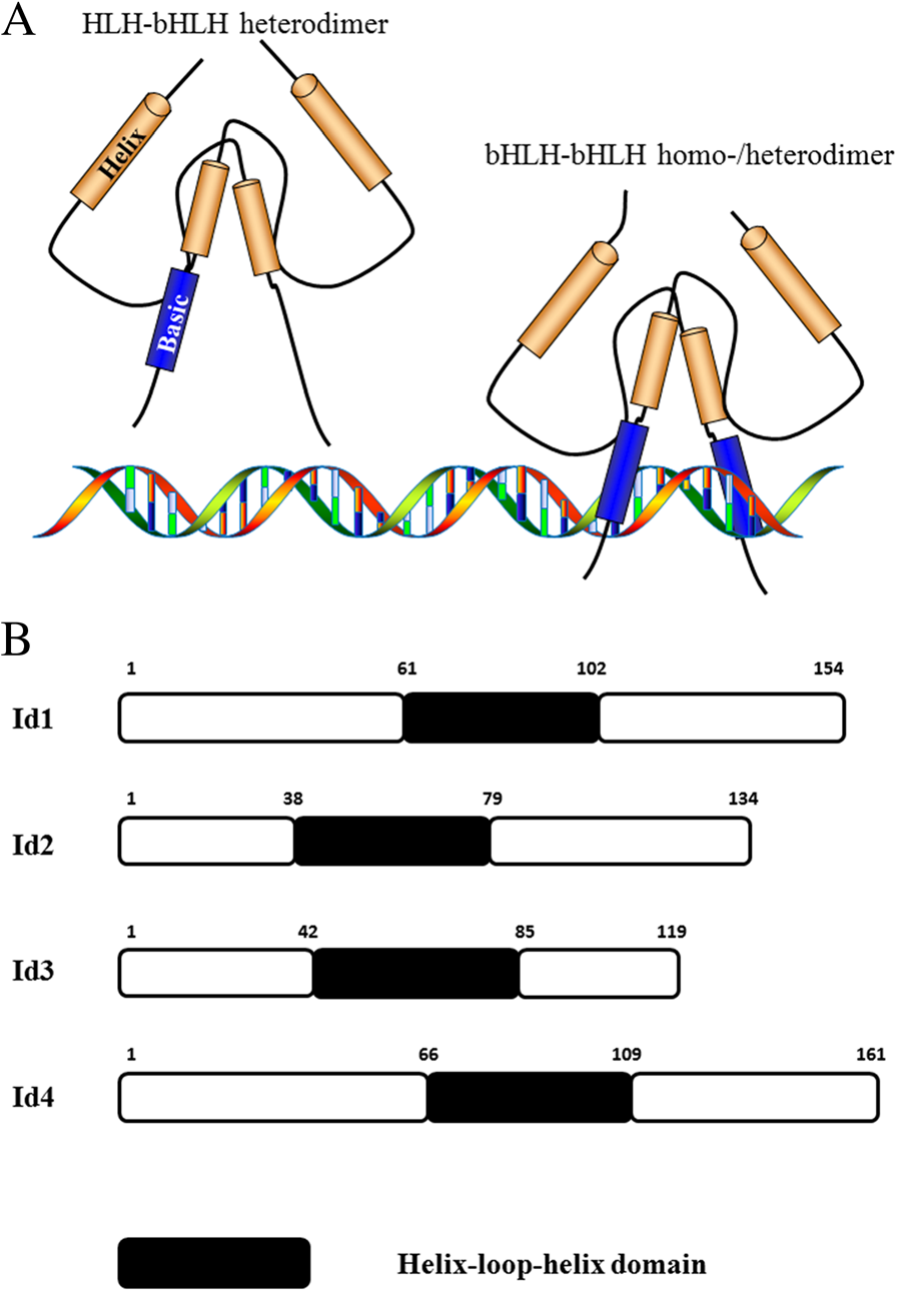
A schematic representation of Id proteins is shown. **a**, The lack of the ‘basic’ DNA-binding domain makes it impossible for HLH/bHLH heterodimers to bind to DNA directly. **b**, The black box represents the helix-loop-helix domain, which is important for Id dimerisation with other E proteins

Id proteins, encoded by the Id gene family, consist of Id1, Id2, Id3, and Id4 (Fig. 1). In vertebrates, Id gene family members encode transcription regulators that contain a highly conserved HLH domain but lack a DNA binding domain; therefore, these regulators are unable to bind to DNA directly. Id proteins inhibit gene expression and regulate growth and development by binding to and isolating the ubiquitously expressed E-proteins, Tcf3, Tcf4, and Tcf12 [11–16]. Ids are also identified as differential inhibitors according to the effects of differentiation repression [17, 18]. Ids are essential for skeletal myogenesis [19] and cardiac development [20]. Ids also play key roles in the differentiation and proliferation of several cell lineages (such as neural precursor cells [21, 22], B cells [23], regulatory T cells [24], helper T cells [25], retinal progenitor cells [26], cortical precursors [27], myeloid progenitor cells [17, 28], keratinocytes [29, 30], pulmonary artery smooth muscle cells [31], and hair cells during mechanoreceptor organ development [32]), entrainment and operation of the circadian system [33, 34], proliferation of biliary epithelial cells and liver regeneration [35], corpus luteum and vascular remodelling [36], and induction of hormone secretion in melanotrophs [37]. Ids are also involved in multiple diseases, especially in cancers of various organs [38–42].

### Ids in heart development

#### Id1

Nearly three decades ago, Id1, the first Id gene, was named after the protein’s ability to inactivate the DNA binding of bHLH transcription factors [43]. In mice, Id1, Id2 and Id3 were reportedly detected in the endo- and epicardium from embryonic day (E) 10.5 through 16.5 [44]. According to a recent study, Id1 is expressed in the most proximal epiblast region of embryos during gastrulation (E6.5–E7.25) and in the anterior lateral mesoderm containing undifferentiated cardiac precursors [45].

Structurally, the depletion of *Id1*, *Id2* or *Id3* cannot lead to developmental abnormalities, but double or triple *Id* knockout embryos (*Id1/Id2*, *Id2/Id3*, *Id1/Id3* or *Id1/Id2/Id3*) exhibit severe cardiac defects including valvular and septal defects, outflow tract atresia, impaired ventricular trabeculation and thinning of the compact myocardium layers; the embryos die at mid-gestation [46] (Table 1). Functionally, the expression of Id1 induces apoptosis in cardiac myocytes [47]; however, embryos with *Id1–3* deficiency display reduced cell proliferation in the ventricular compact layer. Valvular interstitial cells are yielded from endocardial cells contributing to the cushions of the atrioventricular canal and outflow tract [48, 49]. Using RNA-seq, DeLaughter et al. identified Id1 as a candidate gene important for endocardial epithelial-to-mesenchymal transformation in the chick and mouse embryo [50], which explains the phenotypes of valvular defects and outflow tract atresia seen in *Id1* null mice. The expression of the cardiac specific markers, Gata4, α-MHC [51] and Isl1 [52], are upregulated in P19CL6 cells transfected with *Id1* during cardiac differentiation and Id1 can promote proliferation of these cells in vitro [53]. Similarly, Id1 is needed for normal cardiogenic mesoderm differentiation in mouse embryonic stem cells (ESCs) and is sufficient to direct ESCs to differentiate towards the cardiac mesoderm [45]. Thus, despite functional redundancy, Id1 regulates differentiation of cardiac precursors and is involved in proliferation and apoptosis of cardiomyocytes.

**Table 1:** Developmental phenotypes of Id-knockout animal model

Model	Species	Cardiac Phenotype	Reference
*Id1* konckdown by siRNA	Chick and mouse	Decreased endocardial epithelial-to-mesenchymal transformation	50
*Id2* ^*−/−*^	Mouse	Atrioventricular septal defects and membranous ventricular septal defects in *Id2* null perinatal death, left bundle branch block, indicative of ventricular conduction delay in surviving mice	54, 55
*Id4* ^*−/−*^	Zebrafish	Retrograde blood flow at the atrioventricular canal, indicating defects in atrioventricular valve function	61
*Id1* ^*−/−*^ *Id3* ^*−/−*^ *; Id1* ^*−/−*^ *Id2* ^*−/−*^ *; Id1* ^*−/−*^ *Id2* ^*+/−*^ *Id3* ^*−/−*^ *; Id1* ^*+/−*^ *Id2* ^*+/−*^ *Id3* ^*−/−*^	Mouse	Ventricular septal defects, impaired ventricular trabeculation, thinning of the compact myocardium, and outflow tract atresia at E11.5 to E13.5	46
*Id1* ^*−/−*^ *Id2* ^*−/−*^ *Id3* ^*−/−*^	Mouse	Reduced heart size by 40 to 60%, defects in auriculoventricular dissociation and thinner ventricular compact layer from E9.5	46
*Id1* ^*−/−*^ *Id2* ^*−/−*^ *Id3* ^*−/−*^ *Id4* ^*−/−*^	Mouse	Heart tube-forming region missed at cardiac crescent stages	45
*Id1*^*f/f*^*Id3*^*−/−*^ or *Id1*^*f/−*^*Id3*^*−/−*^; *Tie2-Cre*	Mouse	Fibrotic vasculature, cardiac enlargement, ventricular septal defects and decreased cardiac function	70

*E* embryonic day, *Id* inhibitor of DNA binding, *siRNA* small interfering RNA, *Tie2* TEK receptor tyrosine kinase

#### Id2

Id2 mRNA can be detected in the extraembryonic but not in the embryonic ectoderm from E6.5 onwards. In the developing heart, Id2 expression can be seen in the developing cardiac neural crest, outflow tract, and inflow tract, as well as in the neurons surrounding the developing aorta, pulmonary artery, the epicardium and the endocardium from E10.5, but it is absent in the myocardium [46]. Id2 is expressed in the nascent atrioventricular bundle at E12.5 and in the bundle branches at E16.5 [54].

Although no cardiac phenotype was mentioned in previous studies, Moskowitz et al. found that more than 20% of *Id2* null mice died, and atrioventricular septal defects and membranous ventricular septal defects were observed in these mutant perinatal deaths [55] (Table 1). In addition to its significant function in cardiogenesis, which has been demonstrated using single or multiple Ids knockout animal models in vivo and by using ESCs in vitro (*Id2*^*−/−*^, *Id1*^*−/−*^*Id2*^*−/−*^, or *Id1*^*−/−*^*Id2*^*+/−*^*Id3*^*−/−*^), structurally [56, 57] Id2 plays a key role in the specification of ventricular myocytes into the ventricular conduction system lineage. Electrocardiography on Id2-null mice displays ventricular conduction delay, with a widened QRS complex (RsR’ pattern) in lead I, aVL, and V6, indicative of a left bundle branch block. Histologically, the atrioventricular bundle and left bundle branch seem normal but display reproducible patterning abnormalities [54] (Table 1).

#### Id3

Expression of Id3 can be found in both the embryonic ectoderm and the extraembryonic endoderm at E5.5. From mid-gastrulation, Id1 and Id3 expression exists in partially overlapping patterns in the endocardial cushion (EC) mesenchyme and in the epicardium and endocardium from E10.5; it persists in the endocardium, endothelium, epicardium and cardiac valves until postnatal day 7 [46, 58].

Because of the functional overlap, single *Id3* knockout mice do not show any phenotype during the developmental process [46], which complicates the elucidation of the underlying functions.

#### Id4

Compared with the expression patterns of the other three Id genes, Id4 expression differs from the widespread expression of Idl, Id2, and Id3 in the embryo [44, 59, 60]. Id4 is absent from heart and functionally isolated [44, 46]; thus, it used to be considered irrelevant to heart development.

Until recently, Id4 was found to be expressed in the developing atrioventricular canal endocardium and in the adult atrial chamber in zebrafish embryos during atrioventricular valve formation. *Id4*^*−/−*^ embryonic hearts exhibit impaired atrioventricular valve function (retrograde blood flow from the ventricles to the atria) and reduced endocardial cells contributing to the AV valves [61] (Table 1). To further uncover the potential function of Id genes in early mammalian heart formation, Cunningham et al. generated an Id1–4 quadruple genetically ablated mouse model and observed an absence of heart tube formation at E8.25, when the heart tube should have normally formed, in contrast with Id1–3 triple mutants [45] (Table 1). However, a heart tube can still be formed with only one Id4 wild-type allele, indicating the crucial role of Id4 in early heart formation.

### Regulators of id gene expression

#### BMP signalling pathway

Heart development is a multistep process that involves precise regulation by multiple signalling pathways during embryogenesis, such as bone morphogenetic proteins (BMP) [62, 63] and their downstream targets, Ids. Crossveinless-2, a BMP binding protein [64], is expressed in P19 cells earlier than the cardiac transcription factors Nkx2.5 and Tbx5 and serves as an inhibitor for BMP signalling. Crossveinless-2 can bind to BMP and antagonize its activity, inhibiting the phosphorylation of the Smad1/5/8 complex and downregulating Id1 expression, consequently increasing the induction of cardiac cells [65]. Similarly, the p204 protein enables the differentiation of P19 cells to cardiomyocytes by overcoming inhibition by Id proteins [66].

**Fig. 2:**
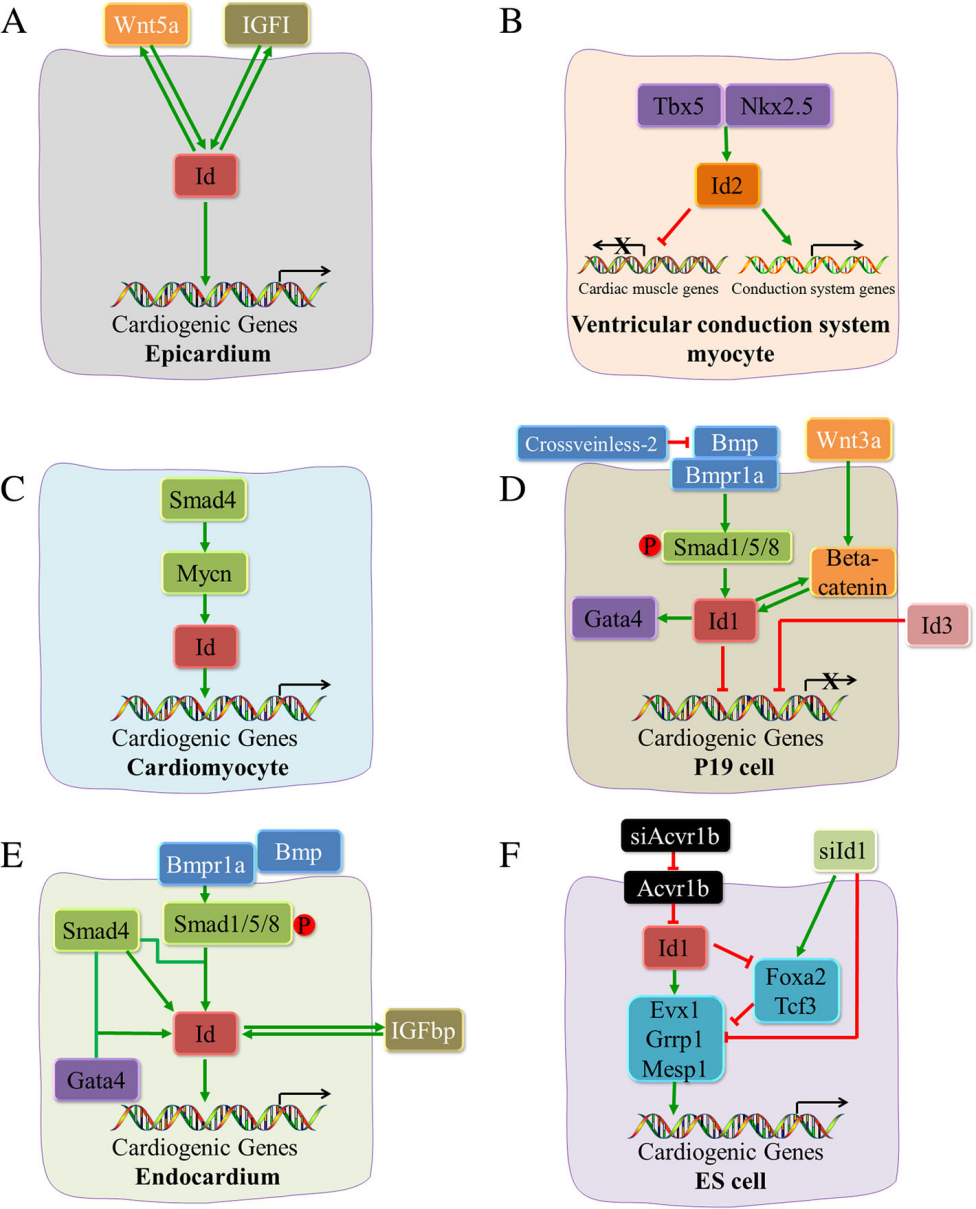
The evidence to date of the differing regulation of Ids in epicardium (**a**), ventricular conduction system myocytes (**b**), cardiomyocytes (**c**), P19 cells (**d**), endocardium (**e**) and ES cells (**f**) is shown

In another study, a *Tie2-Cre* mouse was crossed with a BMP receptor type 1a (BMPR1a) floxed mouse to generate a cKO model. In *BMPR1a-cKO* hearts, atrioventricular valves and adjacent septa failed to form, and expression of Id1/3 was absent from the embryonic atrioventricular canal (AVC) region, suggesting that Bmpr1a expression is needed for AV valve formation and Id1/3 expression in this area [67] (Fig. 2). Activin receptor type-1b (Acvr1b), also named Alk-4, functions as a transducer of activin in the TGF-beta signalling pathway and plays an important role in early endoderm formation [68–71]. The knockdown of Acvr1b using siAcvr1b induces Id1/3 expression in ESCs. As Id1/3 expression increases, early cardiogenic mesoderm markers (Evx1 and Mesp1) [8, 72, 73] are upregulated, and cardiogenic mesoderm formation is induced. Ids are not able to bind to target genes directly; Cunningham et al. demonstrated that this induction of cardiac mesoderm is directed by repressing two mesoderm formation inhibitors—Tcf3 and Foxa2 [74, 75] as well as by activating Evx1, Grrp1, and Mesp1 [45] (Fig. 2).

Smad4 is believed to be a central and essential factor regulating Bmp signalling [76]. After binding to signal ligands, Bmp receptors promote the phosphorylation of Smad1/5/8. With the combination of phosphorylated Smad1/5/8 and Smad4, this complex is able to regulate downstream transcription regulators involved in cardiac development, such as Ids [77]. Smad4 cooperates with Gata4 to activate the *Id2* promoter and regulate cardiac valve development, while mutations in *Smad4* and/or *Gata4* abrogate this activity and lead to endocardial cushion followed by atrioventricular septal defects [55] (Fig. 2). As a direct downstream target transcription factor of Smad4 playing various roles during cardiogenesis and during the development of multiple organs [77], Mycn is indispensable to ventricular wall morphogenesis. Mycn enhances cardiomyocyte proliferation through the regulation of Id2 expression. The cardiomyocyte specific knockout of *Mycn* downregulates the expression of Id2 and reduces cell proliferation in mutant ventricles [78].

#### Wnt signalling pathway

Wnt signalling is required for heart development [79–82]. Injection of ESCs into preimplantation Id KO embryos prevents cardiac defects and corrects 85% of misregulated gene expression profiles throughout the heart due to upregulated expression of Wnt5a in epicardial cells [83]. Furthermore, in P19CL6 cells transfected with Id1, cardiac specific markers, such as Gata4, α-MHC and Isl1 as well as the Wnt signalling pathway components Wnt3a and β-catenin [84] are upregulated and cardiac differentiation and proliferation are induced, while treatment with LiCl (lithium chloride) or Wnt3a upregulates Id1 expression in the same cell lineage, which is indicative of a positive feedback loop between Id1 and Wnt signalling [53] (Table 2, Fig. 2).

**Table 2:** Developmental function and regulation of Ids in different cell lineage in vitro

Cell type	Treatment	Function	Regulation	Reference
P19CL6 cell	Id1/Id1 siRNA transfection	Promote/inhibit differentiation toward cardiomyocyte and cell proliferation	Up−/down- regulate Gata4, α-MHC, and Isl1	53
mESC	Id1 siRNA transfection	Inhibit cardiogenic mesoderm differentiation	Downregulate Kdr, Mesp1, Snai1, and Cdh11	45
mESC or hESC	Id1 transfection	Direct ESCs to differentiate toward the cardiogenic mesoderm	Upregulate Evx1, Grrp1, Mesp1, and Kdr, inhibit Tcf3 and Foxa2 expression	45
P19 cell	Id3 transfection	Inhibit cardiomyocyte differentiation	Inhibite the Expression of the Gata4, Nkx2.5, and MHC, such inhibition can be rescued by p204 protein	66
P19 cell	Crossveinless-2	Promote cardiomyocyte differentiation	Inhibit Smad1/5/8 activation and Id1 expression, enhance expression of T, Mesp1, Nkx2.5 and Tbx5	65

α-MHC, α-myosin heavy chain; Cdh11, cadherin 11; Evx1, even-skipped homeobox 1; Foxa2, forkhead box A2; GATA4, GATA binding protein 4; Grrp1, glycine/arginine rich protein 1; Kdr, kinase insert domain receptor; Isl1, ISL LIM homeobox 1; m/hESC, mouse/human embryonic stem cell; Mesp1, mesoderm posterior bHLH transcription factor 1; Nkx2.5, NK2 homeobox 5; Smad1/5/8, SMAD family member 1/5/8; siRNA, small interfering RNA; Snai1, snail family transcriptional repressor 1; T, T-box transcription factor T or brachyury; Tbx5, T-box transcription factor 5; Tcf3, transcription factor 3

The inactivation of Id4 in zebrafish embryos causes the downregulation of multiple genes crucial for AV canal and AV valve formation, including *spp1*, and elevates Wnt/β-catenin signalling to delay the maturation of valvular cells through the inhibition of TCF activity [61].

#### IGF signalling pathway

In an endocardium- and endothelium-specific Id1/3 conditional knockout (cKO) mouse model (*Tie2-Cre*) [85], cardiac enlargement, and ventricular septal defects were observed in neonatal mice, while fibrotic vasculature and decreased cardiac function were observed in adult ones. Insulin-like growth factor binding protein-3 (IGFbp3) [86], a suppressor of Id proteins, can rescue and reverse gene expression profiles in Id1/3 cKO hearts [87].

To rescue the developmental defects caused by Id1–3 KO, ESCs were injected into preimplantation Id KO embryos. ESCs could partially rescue heart defects through two secreted factors, insulin-like growth factor I (IGFI) [88] and Wnt5a [89] (Fig. 2). IGFI expression overlaps with Id, promotes the proliferation of cardiomyocytes [90], and is downregulated in Id KO epicardial cells. IGFI from ESCs can be released into the Id KO embryos, reversing some of the cardiac defects [83]. However, the reversal is not completely effective.

#### Cardiac transcription factors

In the ventricular conduction system, both in vivo and in vitro analyses confirmed that *Nkx2.5* and *Tbx5*, two key cardiac transcription factor genes, are necessary for Id2 expression in the conduction system cardiomyocytes. *Id2* has a functional binding site in its promoter for *Tbx5* and is a downstream regulatory target of *Tbx5* and *Nkx2.5*.This transcriptional network controls the differentiation balance of the conduction system by inhibiting cardiac muscle gene expression and promoting gene expression specificity pointing to the ventricular conduction system [54] (Fig. 2).

### Ids in cardiovascular diseases and regenerative medicine

#### Congenital heart disease

Congenital heart disease (CHD) is a common malformation related to multiple congenital anomalies, and the mutation of key gene loci or fragments in the development of the heart plays an important role in the occurrence and development of such diseases. Increasing evidence has shown the association of the abnormal expression of Id genes with CHD in animal models [46, 87]. According to Molck’s study, clinically significant copy number variations (CNVs≥300 kb) were detected in patients with congenital heart diseases. ID2 (located in 2p25.1), one of the genes known to participate in cardiac development, was found inside the CNV region of a patient suffering from atrial and ventricular septal defects, pulmonary atresia, and transposition of the great arteries (14,838 kb duplication in 2p24.3-p25.3), which indicates the likely association of ID2 gene defects in human CHD [91].

#### Arrhythmia

The cardiac conduction system functions to initiate and propagate electrical impulses to maintain sequential and effective myocardial contraction. Disorders of the cardiac electrophysiological system are often at the origin of arrhythmias. Id2 expression has been detected in the atrioventricular bundle and bundle branches of mice, and it regulates the differentiation of cardiac precursors towards conduction system cell lineage; Id2-deficient mice show intraventricular conduction delay [54]. Id2 is not only involved in the development of the ventricular conduction system in rodents but also affects electrical signalling in the human atrium. A novel genome-wide association was identified with PR interval, a measure of atrial depolarization and atrioventricular conduction, and a single nucleotide polymorphism (SNP) at ID2 (rs6730558) was confirmed to be associated with prolonged PR interval in Asian, African and European populations. Such a change may lead to atrial fibrillation, heart failure and cardiac mortality [92].

#### Coronary artery pathology

Coronary artery pathology is the leading cause of death worldwide. In addition to adverse lifestyles, genetic variation plays an important role in the occurrence and progression of coronary artery pathology [93]. In humans, ID3 and its SNP (rs11574, related to carotid intima-media thickness) have been demonstrated to be associated with coronary heart disease, as measured by coronary artery calcium, a predictor of coronary disease burden [94], and with atheroma burden by intravascular ultrasound [95] in non-Hispanic White, African American, and Hispanic populations [96, 97]. A meta-analysis on five datasets from the GEO series provides further evidence that ID3 is associated with coronary heart disease [98]. The finding that the ID3 gene is associated with coronary artery pathology can be supported by the fact that Id3 is an atheroprotective transcription regulator which functions to regulate B cell homing and B cell–mediated protection from early atherosclerosis. Furthermore, ID3 reduces atherosclerosis formation [99–101], in addition to T cell differentiation and maturation, which has been demonstrated as playing an important role in atherosclerosis [24].

#### Heart regeneration

Cardiac regeneration has been considered as the most thorough and promising treatment for mocardial injury due to various causes, but this strategy is limited by the extremely low proliferation capacity of mature myocardial cells in post-mitotic state. [102–104]. It has been proposed that fetal cardiomyocytes and progenitor cells have could be used for the development of a potential cell-based therapy, owing to their proliferative stem cell characteristics [105, 106]. The possibility of clinically effective therapies for myocardial damage using primary fetal cardiomyocytes is supported by the current evidence demonstrating that Id1, Id2, and Id3 have implications for the maintenance of the proliferative capacity of human fetal ventricular cardiomyocytes [107]. Another option for inducing cell proliferation and heart regeneration is to reprogram cardiomyocytes into the cell cycle via the delivery of specific stimulators is another option to induce cell proliferation for heart regeneration. A cocktail of three mitosis-related genes, FoxM1, Id1, and Jnk3-shRNA, was delivered to induce cardiomyocytes to re-enter the cell cycle and undergo mitosis in vitro*.* This method successfully increased the proliferation of cardiomyocytes in vivo and reversed cardiac dysfunction after myocardial infarction [108].

Id1 also plays an important role in maintaining the growth activity of embryonic stem cells. Embryonic stem cells provide an indispensable resource for the development of cell-based regenerative medicine [109]. Three genes in chromosome 20q11.21, *ID1*, *BCL2L1*, and *HM13*, are related to culture adaptation of human ES cells [110], thereby providing a strong growth advantage in ES cells, which is highly likely to have a positive effect on subsequent cell differentiation, cardiomyocyte renewal, and heart regeneration.

#### Future perspectives

Single-cell RNA sequencing, a disruptive technology to explore differential gene expression among single cells, has been widely used in development research in multiple organs, including the heart [111–113]. By tracking and sequencing the gene expression of individual Id-expressing cells at different stages, we can fully understand the physiological function of these genes in the process of cardiac development. Similarly, the use of cell classification technology to sequence cell lineages with different fate determinations at the same development stage and compare the expression heterogeneity among cell types horizontally can also deepen the recognition of the distribution and function of Ids.

All four known Id proteins play roles in heart development; Id1–3 share some degree of overlap in terms of expression range and developmental function, but Id4 shows a distinct side [46, 59]. According to Sharma’s study, ID4 heterodimerizes with ID1/2/3 and acts as an inhibitor of ID1–3 proteins; this interaction dependent on helix-loop-helix domain of ID4 [114]. At present this finding has not yet been confirmed in cardiomyocytes. If it is true, then the isolated expression areas of Id4 and Id1–3 can be explained. Likewise, Id4 might be upregulated and compensate for missing functions in Id1/2/3 KO mouse embryos, under circumstance of being repressed by the other three Ids.

The immune system makes an irreplaceable contribution to heart development, composition and function [115]. As previously mentioned, Ids are essential for the differentiation and proliferation of B cells [23], regulatory T cells [24], and helper T cells [25]. Despite no evidence for involvement in the development of coronary arteries as macrophages [116], both B cells and T cells modulate wound healing and tissue repair after myocardial injury [117, 118]. Whether Ids can induce heart repair and regeneration through the regulation of B cell and T cell differentiation and proliferation is well worth further exploration. Additionally, Id1- and Id3-expressing cardiac progenitors give rise to the epicardium, a layer of mesothelial tissue that enfolds the heart; this has been considered as a new source in cardiac repair and regeneration [119], providing another theoretical basis.

Id2 has demonstrated the involvement of the specification of ventricular myocytes into the ventricular conduction system lineage in mice, while depletion of *Id2* leads to intraventricular conduction delay [54]. However, the SNP in ID2 (rs6730558) was identified as related to the PR interval in humans [92], indicative of that Id2 is implicated in normal conduction of cardiac electrical signals through the atrium. Given that the expression of Id2 is exclusive from the sinoatrial node and atrial conduction system [120], we may reasonably reach the hypothesis that Id2 regulates the development of the atrial conduction system through an undiscovered signalling pathway or regulatory network.

Circadian biological activity manifests itself as regular behavior in time. Most organs of eukaryotes have their own biological cycles, and their activities are regulated by the biological clock [121]. Both genetic factors and environmental agents are involved in the regulation of this circadian change in 24-h temporal patterns during the day and at night [122]. The transcriptional repressor Id2, for example, plays a crucial role in circadian rhythms [34]. The rhythm impulse that heart pacemaker cell sends is affected by the circadian clock [123], in addition to the requirement of Id2 for cardiac conduction system development [54], which brings the possibility that Id2 regulates circadian rhythm of the heart rate variability.

## Conclusion

**Fig. 3:**
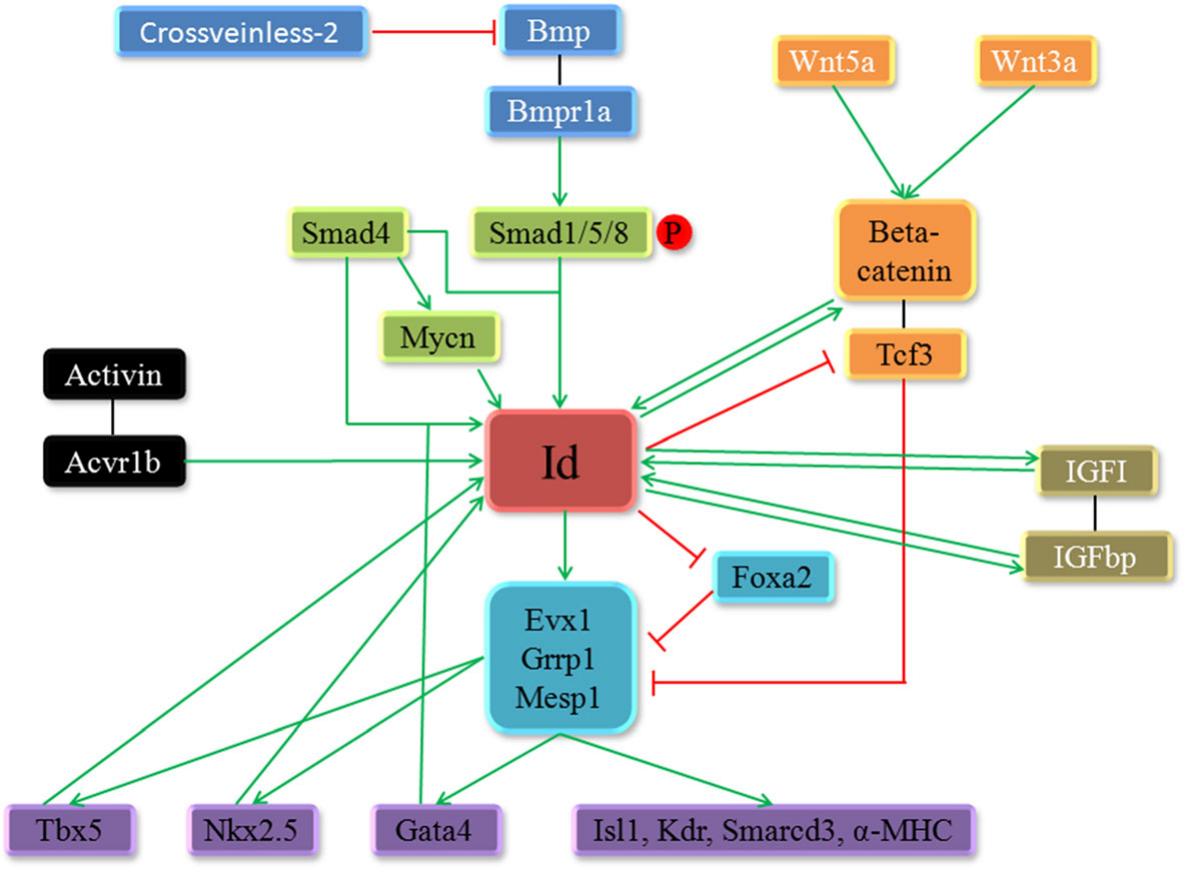
A summary of the regulation network of Ids in heart development is shown. In cardiogenesis, Ids play an important role by regulating the transcription and expression of a variety of key cardiac factors and these regulatory functions are regulated by various signalling pathways and transcription factors. As direct downstream targets of the BMP-Smad signalling pathway, Ids are also regulated by Wnt and IGF signalling pathways. Tbx5 and Nkx2.5, two cardiac transcription factors, also regulate Id to mediate the specification of ventricular myocytes into the ventricular conduction system lineage

In the present article, we provide a comprehensive review of the research to date regarding the role of Id proteins in heart development and the related cardiovascular diseases. During embryonic development, Ids play major roles in early cardiogenesis and during the entire process of the differentiation and proliferation of multiple myocardial cell types, including working cardiomyocytes, endocardial myocardium, epicardial myocardium, and conduction system cells. These physiological functions of Ids are regulated by growth factors and multiple signalling pathways, including Bmp and Wnt, in a cell- and tissue- specific manner to affect heart development (Fig. 2, Fig. 3). The roles of individual Ids in the development of the different tissues and cell lineages of the heart remain to be fully elucidated; however, by tracking and sequencing the gene expression of individual Id-expressing cells at different stages as well as cell lineages with different fate determinations at the same development stage, scientific researchers can fully understand the physiological roles of these genes in the process of cardiac development. Recent studies of Ids have provided tremendous insights into the molecular mechanisms of cardiovascular disease, including congenital structural, coronary, and arrhythmogenic heart disease. Further investigation is needed to determine whether Ids can induce heart repair and regeneration through the regulation of B cell and T cell differentiation and proliferation or though the epicardium that arises from Id1- and Id3-expressing cardiac progenitors. Additionally, Id1 is implicated in the maintenance of the proliferative capacity of human fetal ventricular cardiomyocytes, the reprogramming of mature cardiomyocytes to re-enter cell cycle and regain proliferation capacity, and an increase in ES cell culture adaptability, all of which are indicative of the therapeutic potential for cardiac regeneration.

## Abbreviations

Acvr1b: Activin receptor type-1b
AVC: atrioventricular canal
bHLH: basic helix-loop-helix
BMP: bone morphogenetic proteins
BMPR1a: BMP receptor type 1a
CHD: congenital heart disease
cKO: conditional knockout
CNV: copy number variations
E: embryonic day
EC: endocardial cushion
Id: Inhibitor of DNA binding
IGFbp3: Insulin-like growth factor binding protein-3
SNP: single nucleotide polymorphism
